# Heparin based prophylaxis to prevent venous thromboembolic events and death in patients with cancer - a subgroup analysis of CERTIFY

**DOI:** 10.1186/1471-2407-11-316

**Published:** 2011-07-26

**Authors:** Sylvia Haas, Sebastian M Schellong, Ulrich Tebbe, Horst-Eberhard Gerlach, Rupert Bauersachs, Nima Melzer, Claudia Abletshauser, Christian Sieder, Peter Bramlage, Hanno Riess

**Affiliations:** 1 Institut für Experimentelle Onkologie und Therapieforschung, Technische Universität München, Germany; 2 Abteilung für Angiologie, Klinikum Friedrichstadt, Dresden, Germany; 3 Abteilung für Kardiologie, Klinikum Lippe-Detmold, Detmold, Germany; 4 Phlebology Unit, General Medical Centre, Mannheim, Germany; 5 Max-Ratschow-Klinik für Angiologie, Klinikum Darmstadt GmbH, Darmstadt, Germany; 6 Clinical Research, Novartis Pharma GmbH, Nürnberg, Germany; 7 Biostatistics, Novartis Pharma GmbH, Nürnberg, Germany; 8 Institute for Cardiovascular Pharmacology and Epidemiology, Mahlow, Germany; 9 Medical Clinic Hematology/Oncology, Charité Campus Virchow Klinikum, Augustenburger Platz 1, Berlin, 13353, Germany

## Abstract

**Background:**

Patients with cancer have an increased risk of VTE. We compared VTE rates and bleeding complications in 1) cancer patients receiving LMWH or UFH and 2) patients with or without cancer.

**Methods:**

Acutely-ill, non-surgical patients ≥70 years with (n = 274) or without cancer (n = 2,965) received certoparin 3,000 UaXa o.d. or UFH 5,000 IU t.i.d. for 8-20 days.

**Results:**

1) Thromboembolic events in cancer patients (proximal DVT, symptomatic non-fatal PE and VTE-related death) occurred at 4.50% with certoparin and 6.03% with UFH (OR 0.73; 95% CI 0.23-2.39). Major bleeding was comparable and minor bleedings (0.75 vs. 5.67%) were nominally less frequent. 7.5% of certoparin and 12.8% of UFH treated patients experienced serious adverse events. 2) Thromboembolic event rates were comparable in patients with or without cancer (5.29 vs. 4.13%) as were bleeding complications. All cause death was increased in cancer (OR 2.68; 95%CI 1.22-5.86). 10.2% of patients with and 5.81% of those without cancer experienced serious adverse events (OR 1.85; 95% CI 1.21-2.81).

**Conclusions:**

Certoparin 3,000 UaXa o.d. and 5,000 IU UFH t.i.d. were equally effective and safe with respect to bleeding complications in patients with cancer. There were no statistically significant differences in the risk of thromboembolic events in patients with or without cancer receiving adequate anticoagulation.

**Trial Registration:**

clinicaltrials.gov, NCT00451412

## Background

Patients with cancer have a sixfold increased risk of venous thromboembolism (VTE) compared to those without [1,2], and active cancer accounts for about 20% of all new VTE events occurring in the community [3]. The prevention of VTE in patients with cancer is important, not only because cancer patients have a particularly high risk for VTE, but also because treatment of VTE may be less effective, associated with more bleeding complications and associated with a significant reduction in survival [4-9].

Given the known increased risk of VTE in hospitalized patients with cancer, current guidelines [10-13] recommend risk-stratified thromboprophylaxis for cancer patients at hospital admission with either unfractionated heparins (UFH), low molecular weight heparins (LMWH) or Fondaparinux. Both the European Society for Medical Oncology (ESMO) [14] and the Association of the Scientific Medical Societies (AWMF) in Germany [11] recommend prophylaxis for hospitalized patients with cancer. While ESMO equally recommends LMWH, UFH and Fondaparinux, the German guideline prefers LMWH over the other options based on extrapolations from three placebo controlled randomized trials with LMWH, in which between 5 and 15% of patients had cancer at baseline [11,15-18]. Beyond these data there are randomized controlled trials in cancer patients [19-22] which have demonstrated a prolongation of overall survival with the addition of LMWH, even in patients with advanced disease, but neither rates of VTE nor bleeding complications differed between groups [23]. The German guidelines state that additional studies are needed to resolve this controversy and to clarify which anticoagulant regimes are most likely to be beneficial [10].

To further explore this issue we did a post-hoc analysis of the ***CERT****opar****I****n ****F****or thromboproph****Y****laxis in medical patients *(CERTIFY) study [24], a trial which included 3,239 hospitalized medical patients of at least 70 years and an expected significant decrease in mobility expected for at least 4 days, of which 274 had cancer at hospital admission. CERTIFY has demonstrated non-inferiority of the LMWH certoparin 3,000 U anti-Xa o.d. versus UFH 5,000 IU t.i.d. for the prophylaxis of venous thromboembolism in acutely ill, non-surgical patients aged ≥70 years. We aimed to compare VTE risk and bleeding complications in 1) patients with cancer receiving either LMWH or UFH and 2) patients with or without cancer.

## Methods

We performed a post-hoc subgroup analysis of CERTIFY trial on patients with a diagnosis of co-morbid cancer. Patients had been randomized to receive either 3,000 U anti Xa OD certoparin (Mono-embolex^®^, Novartis Pharma GmbH, Nürnberg, Germany) or 5,000 IU UFH t.i.d. (Liquemin^® ^N 5000, Hoffmann-LaRoche AG, Grenzach-Wyhlen, Germany) in a double-blind fashion [24,25]. The protocol was approved by the ethics committee of Berlin (Landesamt für Gesundheit und Soziales Berlin) and confirmed by local institutional review boards as required by local regulations. All patients provided written informed consent.

Exclusion criteria for CERTIFY were immobilization longer than three days prior to randomization; immobilization due to cast or fracture; expected major surgical or invasive procedure within three weeks following randomization; patients with severe sepsis or need for ventilatory support (permitted were continuous positive airway pressure, oxygen mask etc.); LMWH or UFH longer than 48 hours in the five days prior to randomization; indication for anticoagulation or thrombolysis; life expectancy less than six months or illness with very high acute mortality (> 30%); acute symptomatic DVT/PE; acute or history of heparin induced thrombocytopenia type II (HIT-II); acute or history of non-hemorrhagic stroke (< 3 months); hemorrhagic stroke or intracranial bleeding (< 12 months); acute or ongoing intracranial disease; high risk of gastrointestinal bleeding; spinal or epidural anesthesia, lumbar punction within the last twelve hours; uncontrolled hypertension; severe liver or renal disease; acute endocarditis; known active retinopathy, intravitreal or other intraocular bleeding.

### Endpoints

The primary efficacy measure for the present analysis, as for the overall CERTIFY study, was the combined incidence of proximal DVT, symptomatic non-fatal PE and VTE related death occurring during the core study (covering the treatment period of 8-20 days after which compression ultrasound sonography was performed). All endpoints were adjudicated by a blinded expert committee. Secondary efficacy measures included each of the components of the primary efficacy measure, the incidence of distal DVT (alone and in combination with proximal DVT), the incidence of symptomatic DVT, the incidence of a combination of proximal DVT, non-fatal PE and death from all causes including PE, the incidence of death from all causes; incidence of documented symptomatic VTE.

### Bleeding complications

Major bleeding was defined as fatal bleeding, clinically overt bleeding associated with a fall of the hemoglobin concentration greater than 2 g/l compared to the baseline hemoglobin concentration, clinically overt bleeding that required transfusion of two or more units of packed red cells or whole blood, symptomatic bleeding in a critical area or organ (intracranial, intraspinal, retroperitoneal, and pericardial). All non-major bleeding complications were classified as minor bleeding.

### Statistical analysis

All patients that received at least one dose of study drug were included in the safety analysis (safety population). All patients from the safety population were also included in the intention to treat (ITT) population. The number of patients from the ITT population evaluable for each of the end points is indicated in the respective figures. Point estimates and respective 95% confidence intervals (CIs) were calculated. For details of the statistical analysis of the overall trial see Riess et al. [24]. P-values were determined from 2-sample t-tests for continuous- or from asymptotic odds ratio tests (logistic regression) for binary variables [26]. P-values were determined from univariate logistic regression or from the interaction term of a logistic regression model with factors treatment, subgroup and treatment times subgroup as appropriate.

## Results

### VTE and bleeding complications in cancer patients receiving certoparin or UFH

Cancer patients receiving certoparin (n = 133) and UFH (n = 141) respectively did not differ substantially in any of the documented patient characteristics (Table 1).

**Table 1:** Baseline demographic characteristics for patients with or without cancer (safety population)

	Pts with cancer at admission			Pts without cancer at admission	
	Certoparin (n = 133)	UFH (n = 141)	Total (n = 274)	Total (n = 2965)	p-value vs. pts with cancer
Female (%)	37.6	40.4	39.1	61.0	< 0.0001^^^
Mean age (± SD) (y)	79.7 ± 6.4	78.3 ± 5.8	79.0 ± 6.1	78.8 ± 6.3	0.6922^~^
Mean bodyweight (± SD) (kg)	73.4 ± 14.6	71.2 ± 13.7	72.3 ± 14.2	72.1 ± 15.9	0.8076^~^
Body Mass Index (± SD) (kg/m^2^)	25.9 ± 4.5	25.4 ± 4.6	25.6 ± 4.6	26.6 ± 5.4	0.0041^~^
Reason for hospitalization (%)					
Infections and infestations	23.3	28.4	25.9	27.7	0.5210^^^
Cardiac disorders	14.3	8.5	11.3	23.2	< 0.0001^^^
Respiratory disease	6.8	10.6	8.8	18.0	< 0.0001^^^
Neurologic disease	6.8	6.4	6.6	6.6	0.9791^^^
Gastrointestinal disease	9.0	9.2	9.1	6.3	0.0753^^^
Vascular disease	3.0	6.4	4.7	5.9	0.4454^^^
Renal status					
GFR ≤ 30 ml/min/1.73 m^2^	4.6	2.9	3.7	6.1	0.1106*
30 < GFR ≤ 60 ml/min/1.73 m^2^	52.7	50.0	51.3	52.2	
GFR > 60 ml/min/1.73 m^2^	42.7	47.1	45.0	41.6	
Antiplatelet use					
Yes	51.1	48.9	50.0	52.2	0.4905^^^
No	48.9	51.1	50.0	47.8	
Hospitalization (mean ± SD) (days)	13.0 ± 8.5	12.6 ± 5.2	12.8 ± 7.0	12.3 ± 6.0	0.2500^~^
Immobilization (mean ± SD) (days)	10.7 ± 6.3	10.7 ± 4.5	10.7 ± 5.5	9.8 ± 4.2	0.0038^~^
Mean exposure (± SD) (days)	9.2 ± 3.9	9.1 ± 4.0	9.2 ± 3.9	9.1 ± 3.3	0.6264^~^

Legend: Pts, patients; SD, standard deviation; UFH, unfractionated heparin; ^ Chi-Square Test; * Chi-Square Test comparing only GFR ≤ 30 ml/min/1.73 m^2^; ~ t-Test.

Thromboembolic event rates in patients receiving certoparin (4.50%) or UFH (6.03%) were not significantly different (OR 0.73; 95%CI 0.23-2.39) (Table 2). Event rates for single thromboembolic endpoints were largely comparable between both treatment groups, with total death showing the lowest OR (0.33; 95%CI 0.06-1.64). Although bleeding complications were not statistically different between treatment groups (Table 3), there was a nominal increase in minor bleeding complications in the UFH group.

**Table 2:** Event rates in patients with cancer treated with certoparin or UFH

Pts with cancer at admission	Certoparin		UFH		OR (95%CI)	p-value*
	**n/avail**.	%	**n/avail**.	%		
Thromboembolic events						
Combined endpoint	5/111	4.50	7/116	6.03	0.73 (0.23-2.39)	0.6078
Proximal DVT	5/111	4.50	5/114	4.39	1.03 (0.29-3.65)	0.9656
Symptomatic non-fatal PE	0/129	0	1/128	0.78	-	-
VTE related death	0/131	0	1/132	0.76	-	-
Distal DVT	10/101	9.90	7/106	6.60	1.55 (0.57-4.25)	0.3908
Proximal or distal DVT	12/101	11.88	9/106	8.49	1.45 (0.58-3.61)	0.4213
Symptomatic DVT	1/128	0.78	1/125	0.80	0.98 (0.06-15.78)	0.9866
Death from any cause	2/131	1.53	6/132	4.55	0.33 (0.06-1.64)	0.1743

Legend: Pts, patients; UFH, unfractionated heparin; DVT, deep venous thrombosis; PE, pulmonary embolism; OR, odds ratio; CI, confidence interval, * two-sided p-value for null-hypothesis: difference = 0 or odds ratio = 1.

**Table 3:** Bleeding complications in patients with cancer treated with certoparin or UFH

Pts with cancer at admission	Certoparin		UFH		OR (95%CI)	p-value*
	**n/avail**.	%	**n/avail**.	%		
Patients with cancer at admission						
with major bleeding	1/133	0.75	1/141	0.71	1.06 (0.07-17.13)	0.9669
with minor bleeding	1/133	0.75	8/141	5.67	0.13 (0.02-1.02)	0.0523

Legend: Pts, patients; SD, UFH, unfractionated heparin; OR, odds ratio; CI, confidence interval, * two-sided p-value for null-hypothesis: difference = 0 or odds ratio = 1.

Adverse as well as severe adverse events were comparable in certoparin vs. the UFH treated patients (59.4 vs. 67.4% for AEs and 7.5 vs. 12.8% for SAEs).

### VTE and bleeding complications in patients with or without cancer

Out of a total of 3,239 patients randomized and treated in CERTIFY, 274 had cancer and 2,965 patients no signs of cancer at hospital admission. All were anticoagulated with either certoparin or UFH. Patients with cancer were less frequently female (39.1 vs. 61.0%; p < 0.0001), had a lower body mass index and longer immobilization. With respect to co-morbidity, patients with cancer had less cardiac (11.3 vs. 23.2%; p < 0.0001) or respiratory disorders (8.8 vs. 18.0%; p < 0.0001) (Table 1). For an overview of cancer types see table 4. A total of 5.5% of patients were receiving chemotherapy.

**Table 4:** Cancer types in patients with cancer

	Certoparin (n = 133)		UFH (n = 141)		Total (n = 274)	
	n	%	n	%	n	%
Metastases	21	15.8	18	12.8	49	17.9
Blood	17	12.8	24	17.0	41	15.0
Lung/bronchus	11	8.3	26	18.4	37	13.5
Prostate	23	17.3	12	8.5	35	12.8
Colon/rectum	14	10.5	12	8.5	26	9.5
Breast	16	12.0	6	4.3	22	8.0
Pancreas	14	10.5	8	5.7	22	8.0
Skin	10	7.5	8	5.7	18	6.6
Gastrooesophageal	7	5.3	6	4.3	13	4.7
Urogenital	4	3.0	9	6.4	13	4.7
Liver	4	3.0	8	5.7	12	4.4
Kidney	6	4.5	6	4.3	12	4.4
Gynecological	3	2.3	7	5.0	10	3.6
CNS	4	3.0	5	3.5	9	3.3
Others	4	3.0	3	2.1	7	2.6
Unclassified	9	6.8	8	5.7	17	6.2

Legend: * unknown primary tumour.

Thromboembolic event rates (proximal DVT, symptomatic non-fatal PE and VTE related death) were not significantly different in patients with cancer versus those without (5.29 vs. 4.13%; p = 0.4097) (Table 5). While differences in single thromboembolic endpoints were only minor, the rate of patients dying from any cause was increased in patients with cancer (3.04 vs. 1.16%; p = 0.0136). The rate of major and minor bleeding complications was comparable between both patient groups (Table 6).

**Table 5:** Event rates in patients with and without cancer

All patients	Pts with cancer at admission		Pts without cancer at admission		OR (95%CI)	p-value*
	**n/avail**.	%	**n/avail**.	%		
Thromboembolic events						
Combined endpoint	12/227	5.29	104/2516	4.13	1.29 (0.70-2.39)	0.4097
Proximal DVT	10/225	4.44	98/2516	3.90	1.15 (0.59-2.23)	0.6851
Symptomatic non-fatal PE	1/257	0.39	9/2827	0.32	1.22 (0.15-9.69)	0.8488
VTE related death	1/263	0.38	0/2852	0	-	-
Distal DVT	17/207	8.21	178/2270	7.84	1.05 (0.63-1.77)	0.8495
Proximal or distal DVT	21/207	10.14	218/2280	9.56	1.07 (0.67-1.71)	0.7851
Symptomatic DVT	2/253	0.79	7/2814	0.25	3.20 (0.66-15.46)	0.1488
Death from any cause	8/263	3.04	33/2852	1.16	2.68 (1.22-5.86)	0.0136

Legend: Pts, patients; UFH, unfractionated heparin; DVT, deep venous thrombosis; PE, pulmonary embolism; OR, odds ratio; CI, confidence interval, * two-sided p-value for null-hypothesis: difference = 0 or odds ratio = 1.

**Table 6:** Bleeding complications in patients with or without cancer (safety population)

All patients	Pts with cancer at admission		Pts without cancer at admission		OR (95%CI)	p-value*
	**n/avail**.	%	**n/avail**.	%		
Bleeding complications						
with major bleeding	2/274	0.73	15/2965	0.51	1.45 (0.33-6.36)	0.6254
with minor bleeding	9/274	3.28	101/2965	3.41	0.96 (0.48-1.93)	0.9152

Legend: Pts, patients; SD, UFH, unfractionated heparin; OR, odds ratio; CI, confidence interval, * two-sided p-value for null-hypothesis: difference = 0 or odds ratio = 1.

With respect to patient safety, the number of those experiencing any severity of adverse events was comparable (63.5 vs. 61.2%; OR 1.10 (0.85-1.43)), while serious adverse events and especially death were significantly more common in those with cancer (Table 7). Differences between SAE death rates and those of the efficacy anaylsis are because of a different denominator (intention to treat vs. safety population).

**Table 7:** Safety data for patients with or without cancer (safety population)

All patients	Pts with cancer at admission				Pts without cancer at admission	
	Certoparin (n = 133)	UFH (n = 141)	OR (95%CI)	Total (n = 274)	Total (n = 2965)	OR (95%CI) vs. pts. with cancer
Patients with AEs; n (%)	79 (59.4)	95 (67.4)	0.71 (0.43-1.16)	174 (63.5)	1815 (61.2)	1.10 (0.85-1.43)
Suspected drug relation	5 (3.8)	6 (4.3)	0.88 (0.26-2.95)	11 (4.0)	82 (2.8)	1.47 (0.77-2.79)
Dose adjustment or study drug interruption	1 (0.8)	2 (1.4)	0.53 (0.05-5.88)	3 (1.1)	17 (0.6)	1.92 (0.56-6.59)
Study drug discontinuation	6 (4.5)	7 (5.0)	0.90 (0.30-2.76)	13 (4.7)	115 (3.9)	1.23 (0.69-2.22)
Concomitant medication/non- drug therapy	56 (42.1)	76 (53.9)	0.62 (0.39-1.00)	132 (48.2)	1265 (42.7)	1.25 (0.98-1.60)
Patients with SAEs (n, %)	10 (7.5)	18 (12.8)	0.56 (0.25-1.25)	28 (10.2)	172 (5.8)	1.85 (1.21-2.81)
Deaths from any cause	2 (1.5)	6 (4.3)	0.34 (0.07-1.73)	8 (2.9)	33 (1.1)	2.67 (1.22-5.84)
Suspected drug relation	0 (0)	1 (0.7)	-	1 (0.4)	10 (0.3)	1.08 (0.14-8.49)
Study drug discontinuation	1 (0.8)	3 (2.1)	0.35 (0.04-3.39)	4 (1.5)	62 (2.1)	0.69 (0.25-1.92)

Legend: HIT II, Heparin induced thrombocytopenia; UFH, unfractionated heparin; AE, adverse event; SAE, serious adverse event.

## Discussion

The present subgroup analysis of the CERTIFY trial resulted in the following findings: 1) Thromboembolic events were reduced from 6.03 to 4.50% with the use of certoparin in comparison with UFH, although this did not reach statistical significance. Further there was a nominal but not statistically significant increase in minor bleeding complications as well as all cause death in the UFH group. 2) The rates of venous thromboembolism in cancer patients are comparable with adequate anticoagulation (LMWH or UFH) with no difference in bleeding complications. Serious adverse events and especially all cause death were however significantly more common in those with cancer.

### VTE and bleeding complications in cancer patients receiving certoparin or UFH

Both certoparin and UFH were statistically equally efficacious to prevent thromboembolic events in the present analysis (6.03% with UFH, 4.50% with certoparin; p = 0.6078). This is overall in good agreement with the results of the total CERTIFY trial population in which event rates were 4.52% with UFH and 3.94% with certoparin [24], indicating non-inferiority of certoparin vs. UFH.

The only comparable analysis was a subanalysis of the MEDENOX study, in which enoxaparin (20 or 40 mg) was compared to placebo in the prevention of venous thromboembolism [18]. Venous thromboembolism (between day 1 and day 14) was defined as deep vein thrombosis, clinical suspicion of deep vein thrombosis, (fatal) pulmonary embolism. A total of 18.6% of patients (22 out of 118 patients with cancer) experienced VTE during the observation, but no data were presented on the relative efficacy of enoxaparin vs. placebo in the subgroup of patients with cancer.

Although bleeding complications were not statistically different between treatment groups, there was a nominal decrease in minor (1.50 vs. 6.38%; OR 0.22; 95%CI 0.05-1.06) bleeding complications in the certoparin group. This might somewhat relate to the mode of application, because UFH was administered as a subcutaneous injection three times daily, while certoparin was administered once daily. It appears however to translate into a patient related benefit. Bleeding rates overall were well comparable with previous trials [15-18].

### VTE and bleeding complications in patients with or without cancer

Patients with cancer have a sixfold increased risk of venous thromboembolism (VTE) compared to those without, but this risk increase has been documented for patients without prophylaxis [1,2]. The additional risk can almost be abolished by heparin prophylaxis (UFH or LMWH) as demonstrated in the present analysis, where VTE rates (Incidence of proximal DVT, symptomatic non-fatal PE and death from any cause) were not increased in patients with cancer versus those without (5.29 vs. 4.13%; p = 0.4097), reinforcing the need for an effective prophylaxis. In agreement with data from previous trials [15-18], the bleeding risk and complications rates observed were low, justifying the use of pharmacologic thromboprophylaxis in hospitalized patients with cancer.

### Limitations

Despite the high relevance of the present data for clinical practice, there are some inherent limitations to the present subgroup analysis. 1) There are considerable differences in VTE risk between patients with different cancer types and we have not captured these in sufficient detail to account for differences in patient characteristics, in particular for the comparison of certoparin and UFH. 2) The subgroup of patients with cancer was small compared to the overall sample size of CERTIFY. The power of the analysis was therefore limited in this subgroup as illustrated by numerically large differences between the groups which could not be statistically validated. 3) There is no possibility to explore the absolute efficacy of either heparin in comparison to patients receiving no prophylaxis, but there are a number of studies documenting this [15,19-23].

## Conclusions

Certoparin 3,000 UaXa o.d. and 5,000 IU UFH t.i.d. were equally effective and safe with respect to bleeding complications in patients with cancer. There were no statistically significant differences in the risk of thromboembolic events in patients with or without cancer receiving adequate anticoagulation.

## Competing interests

SH, SMS, UT, HEG, RB, PB and HR have received research support and honoraria for lectures from a number of pharmaceutical companies including Novartis, the sponsors of this study. NM, CA and CS are employees of the sponsor.

## Authors' contributions

SH, SMS, UT, HEG, RB, CA, CS and HR have been involved in the conception and design of the study. CS was responsible for the analysis of data in cooperation with PB. SH and PB SH have drafted the manuscript and all other authors have been revising the article for important intellectual content. All authors have finally approved the version to be published.

## Pre-publication history

The pre-publication history for this paper can be accessed here:

http://www.biomedcentral.com/1471-2407/11/316/prepub
